# Dendritic Cell Subsets in Asthma: Impaired Tolerance or Exaggerated Inflammation?

**DOI:** 10.3389/fimmu.2017.00941

**Published:** 2017-08-09

**Authors:** Heleen Vroman, Rudi W. Hendriks, Mirjam Kool

**Affiliations:** ^1^Department of Pulmonary Medicine, Erasmus MC, Rotterdam, Netherlands

**Keywords:** asthma, dendritic cells, Th2 cells, Th17 cells, airway inflammation

## Abstract

Asthma is a prevalent chronic heterogeneous inflammatory disease of the airways, leading to reversible airway obstruction, in which various inflammatory responses can be observed. Mild to moderate asthma patients often present with a Th2-mediated eosinophilic inflammation whereas in severe asthma patients, a Th17-associated neutrophilic or combined Th2 and Th17-mediated eosinophilic/neutrophilic inflammation is observed. The differentiation of these effector Th2 and Th17-cells is induced by allergen-exposed dendritic cells (DCs) that migrate toward the lung draining lymph node. The DC lineage comprises conventional DCs (cDCs) and plasmacytoid DCs (pDCs), of which the cDC lineage consists of type 1 cDCs (cDC1s) and cDC2s. During inflammation, also monocytes can differentiate into so-called monocyte-derived DCs (moDCs). These DC subsets differ both in ontogeny, localization, and in their functional properties. New identification tools and the availability of transgenic mice targeting specific DC subsets enable the investigation of how these different DC subsets contribute to or suppress asthma pathogenesis. In this review, we will discuss mechanisms used by different DC subsets to elicit or hamper the pathogenesis of both Th2-mediated eosinophilic asthma and more severe Th17-mediated neutrophilic inflammation.

Allergen-activated dendritic cells (DCs) are essential not only for the induction of T helper (Th)-cell differentiation from naïve T-cells in the mediastinal lymph node (MLN) but also to drive pulmonary inflammation during continuous allergen exposure (1). Lung DCs are a heterogeneous cell population that contains two types of conventional DCs (cDCs), e.g., cDCs type 1 (cDC1s) cDC2s. Next to cDCs, the lungs also contain plasmacytoid DCs (pDCs) and under inflammatory conditions, monocyte-derived DCs (moDCs) (2–4). DCs can become activated by allergen exposure and by cytokines secreted by the airway epithelium (5, 6). Activation of DCs requires induction of the pro-inflammatory transcription factor NF-κB, which can be negatively regulated by various proteins including the deubiquitinating enzyme tumor necrosis factor alpha interacting protein 3/A20 (7).

## DC Ontogeny

**Figure 1:**
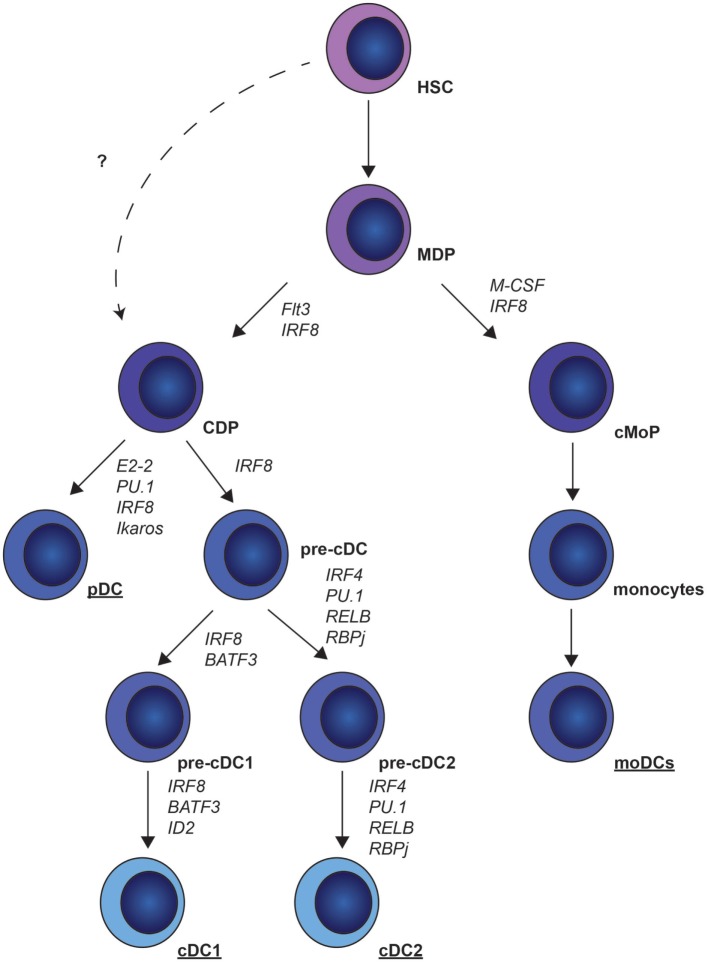
Murine dendritic cell (DC) ontogeny. Common DC precursors (CDP) and common monocytic progenitors (cMoP) develop from myeloid-derived precursors (MDP). CDPs differentiate into plasmacytoid DCs (pDCs) under the control of E2-2, PU.1, interferon regulatory factor (IRF)8 and Ikaros, or pre-conventional DCs (cDCs) under the control of IRF8. Pre-cDCs give rise to pre-cDC1 and pre-cDC2, which finally differentiate into cDC1s and cDC2s. cMoPs give rise to monocytes that can further differentiate into monocyte-derived DCs (moDCs).

Dendritic cells originate from hematopoietic stem cells (HSCs) in the bone marrow (BM). These HSCs differentiate into the macrophage DC progenitors (MDPs) (8), which give rise to common monocyte progenitors (cMoPs) and common DC progenitors (CDPs) (Figure 1). Whether CDPs also develop without the intermediate MDP stage is currently unknown. CDPs give rise to pre-cDCs and pDCs (9). BM pre-cDCs contain pre-cDC1s and pre-cDC2s that are committed to cDC1 and cDC2 development. This indicates that the decision to become cDC1s or cDC2s already occurs in the BM and not in the periphery (10, 11). MoDCs develop from cMoPs (12, 13) (Figure 1).

## Location of Pulmonary DC Subsets

**Figure 2:**
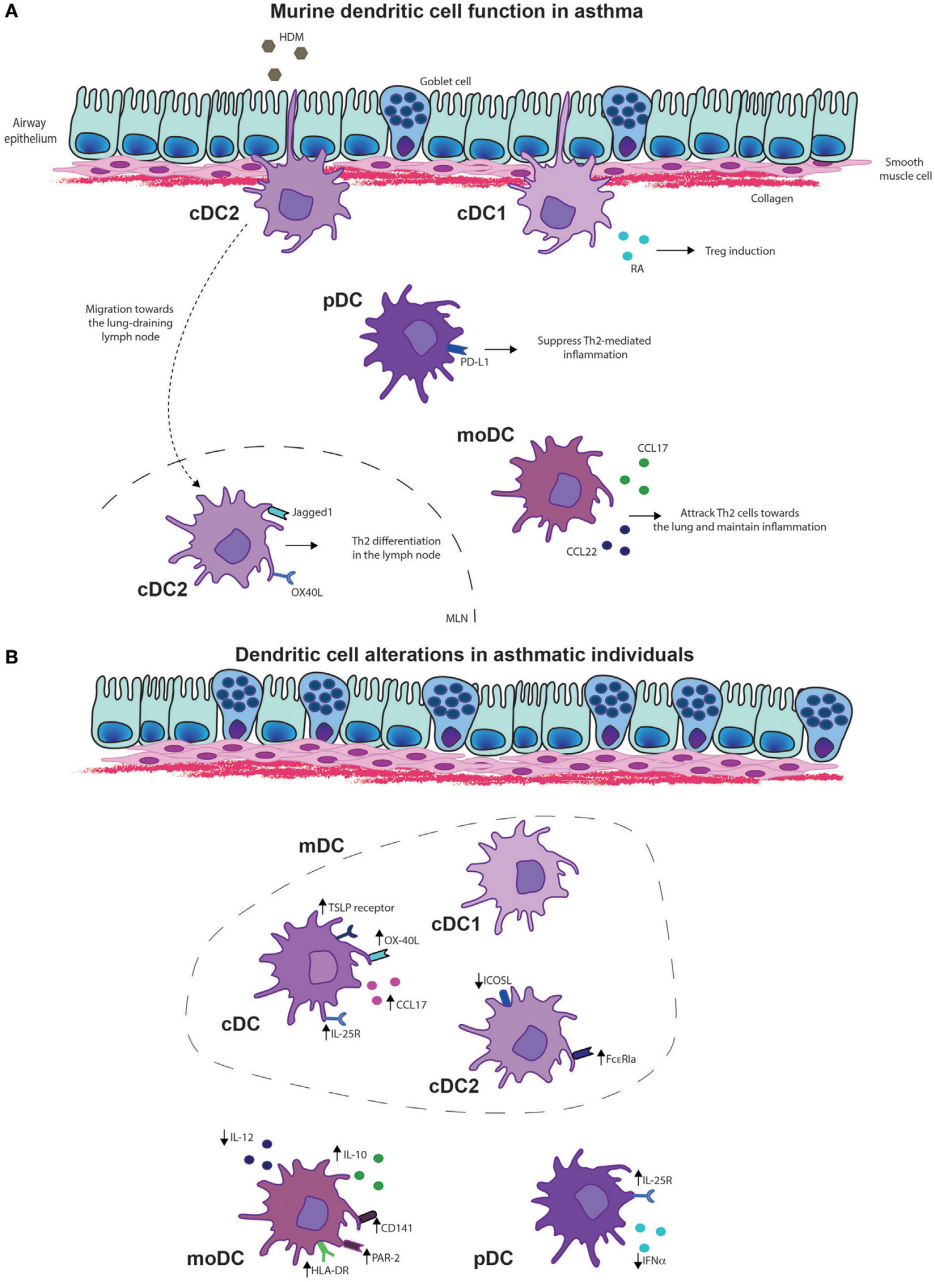
Dendritic cell (DC) functions in asthma. **(A)** Murine DC functions in asthma. Type 2 cDCs are essential for the migration and induction of differentiation of Th2 cells in the lung draining lymph node upon allergen exposure. Monocyte-derived DCs (MoDCs) are important for the chemotaxis of effector Th2 cells toward the lungs by secretion of chemokines CCL17 and CCL22. In asthmatic disease, plasmacytoid DCs (pDCs) suppress Th2-mediated inflammation *via* programmed death-ligand 1 (PD-L1) expression, whereas cDC1s induce regulatory T cells (Tregs) *via* expression of retinoic acid (RA). **(B)** DC alterations in asthmatic individuals. Conventional DCs, including both cDC1s and cDC2s of asthma patients display higher levels of interleukin (IL)-25R, thymic stromal lymphopoietin (TSLP) receptor, OX-40 ligand (OX-40L), and secretion of CCL17. Especially inducible T-cell costimulator ligand (ICOSL) expression in cDC2s of asthmatics is reduced whereas FcεRIa expression is increased in asthmatics that display a Th2 high phenotype. MoDCs of asthmatics display increased expression of human leukocyte antigen-D (HLA-DR), CD141 and protease-activated receptor 2 (PAR-2), and the anti-inflammatory cytokine IL-10, whereas IL-12 production is reduced. pDCs of asthmatics show increased expression of the IL-25R, whereas interferon alpha (IFN-α) secretion was reduced.

Because pulmonary DC numbers are low and until recently multiple markers were needed to specify DC subsets, the number of studies that investigated the location of pulmonary DC subsets during steady state is limited. It is known that cDC1s are located in close proximity of the airway epithelium, whereby their expression of CD103 (alpha integrin) and beta7 integrin (Figure 2A) enables interaction with E-cadherin expressed by epithelial cells. Compared to other DC subsets, cDC1s highly express tight junction proteins, which facilitate the sampling of antigen by extending their dendrites into the airway lumen. cDC1s are also found in the proximity of vascular endothelial cells (14). Most studies that investigated cDC2 localization used CD11b (14–16); however, CD11b is not exclusively expressed by cDC2s and is also highly expressed by moDCs (1). A recent study could distinguish moDCs and cDCs by crossing MacBlue mice (*Csf1r*-ECFP^tg/+^) to *Itgax*-YFP or *Cx3cr1*^gfp/+^ mice, in which cDCs express YFP, using Itgax-YFP mice and monocytes/macrophages express GFP, using *Cx3cr1*^gfp/+^ mice. This study indicated that cDCs are located near the large airways, whereas monocytes and alveolar macrophages are localized in the alveolar space (17). Using MacBlue mice, *in situ* traveling of monocytes and monocyte-derived cells in the lungs was investigated, revealing that monocyte-derived cells are located at the interface of blood vessels and the airways (17, 18). During steady state, the majority of pulmonary pDCs are located in the alveolar interstitium (14, 19); however, pDCs are also found in pulmonary infiltrates in an ovalbumin (OVA)-mediated asthma model (19).

Currently, investigating the localization of DC subsets can be performed with fewer markers, since Guilliams et al. showed that expressions of interferon regulatory factor (IRF)4 and IRF8 are exclusive for cDC2s and cDC1s, respectively, across different organs and species (3). Combining these markers with a universal DC marker such as CD11c should visualize cDC subsets and ease localization studies.

## Murine Conventional Type 1 DCs

### Development of cDC1

cDC1 development is highly dependent on the transcription factor IRF8, as IRF8 drives DC precursor generation (11) and development of pre-cDCs in the BM, and promotes survival of terminally differentiated cDC1s (20). Basic leucine zipper ATF-like transcription factor 3 (BATF3) is implicated in cDC development (21), whereas inhibitor of DNA binding 2 drives terminal differentiation of cDC1s (22) (Figure 1). Ontogeny of cDC1s is regulated by cytokines, as FMS-like tyrosine kinase 3 ligand (Flt3L)-deficient mice completely lack cDC1s in the lungs (1, 23).

### Function of cDC1s in Asthma

cDC1s are well appreciated for their superior cross-presentation of antigens to CD8^+^ T-cells, essential for induction of virus-specific CD8^+^ T-cells and antitumor immune responses (21, 24, 25). However, cDC1s have an inferior capacity to take up allergens compared to other DC subsets (1). Whether cDC1s are also implicated in Th2 skewing in response to allergen exposure remains controversial, as cDC1s are reported to promote, inhibit, or be redundant for Th2 immune responses (1, 26, 27). These differences may be explained by the allergen used, amount of allergen, or the mouse strain used to deplete cDC1s. Besides promoting CD8^+^ T-cell responses, cDC1s are often associated with a tolerogenic function. cDC1s can induce differentiation of Tregs upon house dust mite (HDM) exposure through induction of retinoic acid (RA) and peroxisome proliferator-activated receptor gamma (PPARγ) (26, 28) (Figure 2A). During OVA or HDM-mediated airway inflammation (29) and helminth infections (30), cDC1s can limit Th2 inflammatory responses, emphasizing their tolerogenic potential. Anti-inflammatory properties of *Helicobacter pylori* treatment, which dampens allergic airway disease also depend on cDC1s (31). Furthermore, CD103-deficient mice exposed to an OVA-mediated asthma protocol containing five OVA aerosol challenges developed a more pronounced eosinophilic inflammation indicating their tolerogenic role during Th2-mediated immune responses (29). In contrast, CD103^−/−^ mice that received only a single OVA challenge had decreased eosinophilic inflammation, arguing against the tolerogenic properties of cDC1s. Absence of CD103 did not affect DC migration, but decreased the percentage of allergen-loaded migratory DCs in the MLN (32). Because CD103 can be expressed on both T-cells and cDC1s (33), it is hard to determine which effects are caused by the DCs. However, it is conceivable that cDC1s are essential for allergen uptake at low antigen concentrations. This could explain the decrease in allergen-loaded DCs and the absence of Th2-cell immune responses with only a single OVA challenge. By increasing the allergen exposures, absence of cDC1s can be overcome by protease activity or passive leakage, enabling other DC subsets to access the allergens and migrate toward the MLN.

In addition to their capabilities to suppress Th2-cell differentiation, cDC1s also control Th17 immune responses upon *Aspergillus* infections through secretion of interleukin (IL)-2 (34), indicating that cDC1s maintain homeostasis in the airways. Furthermore, cDC1s are also important for the removal of apoptotic cells, because resolution of airway inflammation is reduced in CD103-deficient mice (29), and cDC1s have been shown to remove apoptotic cells (35).

As it was described that pulmonary cDC1s express Langerin (14), some studies that investigated pulmonary cDC1 function used Langerin-diphtheria toxin (DTR) mice to specifically deplete pulmonary cDC1s (1). However, flow cytometric analysis showed that only a minority of the pulmonary cDC1s expressed Langerin (36), indicating heterogeneity within the pulmonary cDC1 population.

## Murine Conventional Type 2 DCs

### Development of cDC2

In contrast to knowledge on cDC1 development, the transcriptional control of cDC2s is not well characterized. Differentiation of cDC2s from pre-cDCs is regulated by v-rel avian reticuloendotheliosis viral oncogene homolog B (37), PU.1 (38), recombination signal-binding protein 1 for J-Kappa (39–41), and IRF4 (42–44). However, it is unknown during which cDC2 developmental stage these transcription factors are important (Figure 1). Also, the role of the cytokine Flt3L in cDC2 development is controversial, as it has been reported that cDC2 development is both dependent (1) and independent (23) of Flt3L. These differences are likely caused by different methods to distinguish cDC2s from moDCs, leading to cDC2 fractions containing moDCs that develop independent of Flt3L (1). The newly proposed universal gating strategy using IRF4 and IRF8 (3) makes the distinction between DC subsets easier and will help future studies investigating the role of specific transcription factors or cytokines in the development of DC subsets.

### Function of cDC2s in Asthma

cDC2s can take up allergen very efficiently (1, 45), migrate well to the MLN, and induce proper T cell proliferation (1) (Figure 2A). cDC2s are essential for the induction of Th2-cell differentiation in allergen-exposed lungs (1, 45, 46) and possess the capability to induce Th17-cell differentiation in the gut (42, 47). In an HDM-mediated asthma model, cDC2s induced both Th2 and Th17 differentiation (48). HDM can be recognized by various innate receptors on the cell membrane of DCs, including C-type lectin receptors, such as Dectin-2 (49). Differentiation of both HDM-mediated Th2 and Th17 is dependent on Dectin-2-mediated recognition and/or allergen uptake, as both Th2 and Th17 cytokines are reduced in T-cells of Dectin-2-deficient mice (48). cDC2-deficient mice through IRF4 deficiency have reduced Th2 immune responses in the airways upon sensitization in the airways (50) or in the skin (51). Similarly, no eosinophilic inflammation or Th2 differentiation was induced in mice in which IRF4 was depleted in mature DCs, using a different *CD11c*Cre, which did not affect cDC2 cell development (52). This indicates the importance of cDC2s for the induction of Th2 differentiation. Dectin-1 expression on DCs appears to be important for migration, as Dectin-1-deficient cDC2s display lower levels of CCR7, and have lower numbers of migratory cDC2s in the MLN. Furthermore, Dectin-1^−/−^ mice did not develop eosinophilic inflammation, nor did they show induction of Th2 or Th17 cytokines in an HDM-mediated asthma model (53). These findings indicate that Dectin-1 is required for the induction of chemokines and chemokine receptors on cDC2s, enabling migration and T-cell differentiation. cDC2s exclusively express the TNF-superfamily member OX-40 ligand (OX-40L) (54), essential for Th2 cell differentiation, indicating the importance of cDC2s for Th2 differentiation (Figure 2A). In neonatal mice, HDM-induced IL-33 production suppressed IL-12p35 expression and induced OX-40L in cDC2s, driving Th2 differentiation (55).

## Murine moDCs

### Development of moDCs

As their name implicates and stated above, moDCs derive from monocytes. There are two types of monocytes, Ly6C^hi^ and Ly6C^low^ monocytes (56). Ly6C^hi^ monocytes migrate toward inflammatory sites and give rise to Ly6C^hi^ moDCs, Ly6C^low^ moDCs (57), and Ly6C^low^ monocytes (58). MoDCs downregulate Ly6C upon differentiation from monocytes (1). Ly6C^low^ monocytes patrol the vasculature (56, 59) and differentiate into more long-lived Ly6C^low^ moDCs (57). It is suggested that monocytes or moDCs can serve as cDC precursors, in which cDC1s arise from Ly6C^hi^ CCR2^hi^ monocytes, and cDC2s develop from Ly6C^low^ CCR2^low^ monocytes (60).

### Function of moDCs in Asthma

After a primary high dose of HDM in the airways, moDCs accumulate within 48 h in the lungs and peak at 72 h in the MLN (1). HDM and other environmental factors trigger the airway epithelium to secrete chemokines and cytokines (61). Secretion of CCL2 will drive migration of monocytes toward the lungs (62), where they will differentiate into moDCs under the regulation of both CCL2 (1) and colony-stimulating factor 1 (23). MoDCs are efficient in antigen uptake; however, their capacity to drive T-cell proliferation is inferior to cDC2s. Instead, moDCs produce vast amounts of cytokines and chemokines essential for the recruitment and activation of Th2-cells upon HDM exposure (1) (Figure 2A). This indicates that moDCs are dispensable for Th2 differentiation but essential during the effector phase of asthma models, as depletion of CD11b^+^ myeloid cells, which includes monocytes, during allergen challenge drastically reduces eosinophilia (63). Nevertheless, with high antigen dose, moDCs migrate toward the MLN and induce Th2 differentiation in the absence of cDCs upon exposure to HDM (1) or cockroach extract (64). Depletion of migratory cDCs enhances Th2 cell-mediated immune responses in an OVA-alum model (65). Furthermore, absence of Th2-cell-mediated immunity, due to the absence of DCs, can be reverted by an adoptive transfer of monocytes that differentiate into moDCs (66). Likewise, it was shown that systemic administration of BM-derived CD11b^+^ cells efficiently induces Th2-mediated eosinophilic airway inflammation (67). This implicates that at high allergen concentration, moDCs can acquire migratory capacities, induce Th2 differentiation, and thereby drive Th2-mediated immune responses.

## Murine pDCs

### Development of pDCs

Plasmacytoid DCs differentiate directly in the BM from CDPs (68). Differentiation of pDCs depends on Flt3L and signal transducer and activator of transcription 3 signaling, in combination with transcription factors, such as E2-2, IRF8, Ikaros, and PU.1, of which E2-2 is highly specific for pDC development (69–71) (Figure 1).

### Function of pDCs in Asthma

Plasmacytoid DCs are essential for antiviral immune responses as they produce large amounts of interferon alpha (IFN-α) after Toll-like receptor (TLR7) activation (72, 73). In comparison to other DC subsets, pDCs have a limited capacity to take up and present antigens (1, 19, 74, 75). pDCs have a tolerogenic function in asthma, as pDCs induce Treg cell differentiation (76, 77), and depletion of pDCs in Siglec-H-DTR mice increased the proliferation of antigen-specific CD4^+^ T-cells (78). Increase in pDC numbers, as induced by Flt3L treatment, alleviate eosinophilic inflammation, which is reversed upon pDC depletion (79). Programmed death-ligand 1 (PD-L1) expression on pDCs is essential for their suppressive effect, as PD-L1-deficient pDCs could not alleviate allergic airway inflammation, whereas IDO or inducible T-cell costimulator ligand (ICOSL)-deficient pDCs could do so (79) (Figure 2A). Development of HDM-driven allergic asthma can be inhibited by adoptive transfer of pDCs from sensitized donors (80). Different pulmonary pDC subsets have been described, e.g., CD8α^−^β^−^, CD8α^+^β^−^, and CD8α^+^β^+^ pDCs (81). Only CD8α^+^β^−^ pDCs and CD8α^+^β^+^ pDCs have tolerogenic capacities, whereas CD8α^−^β^−^ pDCs display more pro-inflammatory functions upon TLR7 and TLR9 stimulation (81). Specifically, CD8α^+^β^+^ pDCs and CD8α^+^β^−^ pDCs have increased expression of retinal dehydrogenase leading to RA production, resulting in increased Treg differentiation (81).

Plasmacytoid DCs are essential for beneficial effects observed in immunotherapy *via* complement subunit C1q. Administration of C1q reduces airway hyperresponsiveness (AHR) and eosinophilia as efficiently as dexamethasone administration. pDC depletion abrogates the protective effect of C1q (82).

Viral infections are often detected during asthma exacerbations. Viral particles activate DC subsets *via* TLR7, and its expression was decreased in pDCs by allergic inflammation. TLR7-deficient mice displayed reduced IFN secretion, increased virus replication, and increased eosinophilic inflammation and AHR, indicating that impaired TLR7 expression on pDCs by allergic inflammation exaggerates asthma exacerbations (83). Furthermore, pDCs transferred from donors with a respiratory tract syncytial virus (RSV) infection did not provide protection from Th2-mediated inflammation as transferred pDCs from naïve mice did (80). CpG-maturated pDCs are well capable of protecting from eosinophilic inflammation (79), suggesting that altered activation of pDCs affects their function.

## Human Pulmonary DC Subsets

### Transcriptional Development of Human DCs

In human lungs, three different DC subsets have been described, human DC1s, which express BDCA1/CD1c, DC2s, which expresses BDCA3/CD141 and pDCs, which express BDCA2/CD123 (3, 4, 84). Gene expression profiles of human DC1s and DC2s revealed that human DC subsets resembled mouse cDC1s and cDC2s, respectively (3, 85–88). Development of human DCs is also highly dependent on Flt3L, as Flt3L injection drastically increases the number of blood DCs of healthy volunteers (89). Similar to that in mice, differentiation of human pDCs is mediated by E2-2 (90), whereas cDC1 and cDC2 differentiation is controlled by BATF3 (91) and IRF4 (92, 93), respectively.

### Location of Human DC Subsets in the Lungs

BDCA1^+^ cDC2s were increased in the airway epithelium of asthma patients that display a Th2 phenotype, whereas this was not observed in patients without a Th2 profile (94). DCs are increased in the outer wall of the large airways in patients who suffer from fatal asthma (95). These are likely moDCs, as they express XIIIa (95), a coagulation factor also expressed by macrophages in the skin (96). As both moDCs and macrophages are derived from monocytes this suggests an overlapping ontogeny. Unfortunately, lack of lung material containing epithelium and interstitium of both healthy controls and asthma patients complicates research on the localization of DC subset during steady state and in asthmatic lung. Recent consensus regarding universal markers that can identify DC subsets will facilitate the visualization of DC subsets in human organs (3).

### Function of Human DCs in Asthma

Most studies that investigated human DC function, compared pDCs to myeloid DCs (mDCs) that include both DC1s and DC2s. Allergic asthmatics showed increased frequencies of DC1s and DC2s in peripheral blood (97), induced sputum and bronchoalveolar lavage (BAL) upon allergen inhalation compared to controls (98–100). After allergen inhalation, only DC2s migrated toward bronchial tissue (100). Allergen exposure increased the expression of thymic stromal lymphopoietin (TSLP)-receptor but not IL-33-receptor on cultured cDCs from CD34^+^ BM precursors, which are implicated as Th2 instructive cytokine receptors (100). Allergen inhalation induced expression of IL-25-receptor on both cDCs and pDC (101). The costimulatory molecule OX-40L and expression of Th2 chemoattractant CCL17 was higher on cDCs of patients with mild asthma than on cDCs of healthy controls (102) (Figure 2B). In patients who displayed high Th2-cell numbers, a large proportion of airway mucosal DC2s expressed FcεRIa compared to Th2-cell low asthma patients (94) (Figure 2B). This is likely, as high IgE-levels are associated with high Th2-cell numbers, thereby suggesting that IgE increases FcεRIa expression. IgE-bound antigens are rapidly internalized, processed, and presented by DCs to antigen-specific CD4^+^ T-cells (103, 104). DC2s loaded with Dermatophagoides pteronyssinus antigen P1 (Der p1) allergen-IgE immune complexes induced IL-4 and lowered IFN-γ-expression in *in vitro* cocultures with naïve T-cells (105), indicating that allergen-IgE immune complexes promote Th2 differentiation (106). CD86 expression was higher on mDCs of asthmatic children than on mDCs of atopic children. Furthermore, upon LPS stimulation IL-6 production by mDCs of asthmatic children was decreased, compared with mDCs of atopic children (107). The numbers of IL12-producing mDCs were also lower in asthmatic children (108), indicating the presence of a Th2 promoting environment in asthmatic children. cDCs of allergic asthmatics induced increased Th2 differentiation upon stimulation with TSLP and Der p compared to cDCs of controls (109). TSLP-stimulated cDCs of allergic asthmatics and not of controls, induced IL-9 production and PU.1 expression, indicative of Th9 differentiation (109). When Th2 priming capacity of DC1s and DC2s from human blood and lungs were compared, both lung and blood DC1s were superior in Th2 differentiation (110). However, in this study, live-attenuated influenza virus was used to activate DC1s and DC2s, which primarily activates DC1s, as these induce antiviral immune responses (24). The expression of CD141, a marker for DC1s, is increased in blood leukocytes during acute asthma exacerbations on moDCs, but surprisingly not on DC1s (111). This indicates that DC1s or CD141 expression plays an important role in the pathogenesis of asthma. DC2s from allergic rhinitis patients efficiently prime Th2 differentiation (112) and express lower levels of ICOSL, compared to controls (Figure 2B). Blockade of ICOSL in DC2s of controls increases the production of Th2 cytokines, indicating that decreased ICOSL expression on DC2s promotes Th2 differentiation (113). Both human DC1s and DC2s induce Th2 cytokines; however, Th2 differentiation by cDC1s was observed following exposure to live-attenuated viral particles (112). This implicated that during virus infections, cDC1s in asthmatics shift from promoting Th1 immune responses or maintaining tolerance toward a Th2-promoting phenotype.

Allergen inhalation increased pDCs numbers in the airway lumen (98, 114); however, variable results exist whether circulating pDCs differ between asthmatics and healthy individuals (115, 116). In a birth cohort, circulating pDCs predicted respiratory tract infections, wheezing, and asthma diagnosis by 5 years of age (117). pDCs of severe asthmatics also produced less IFN-α following influenza infection, than pDCs of healthy controls did (118) (Figure 2B). Single-cell analysis revealed that characterization of pDCs based on CD123 expression included DC precursors (119, 120). Therefore, these findings need to be revisited whether they truly induced by pDCs.

House dust mite activation of cultured moDCs of HDM-allergic asthma patients expressed higher levels of human leukocyte antigen-D, induced more T-cell proliferation (121), and Th2 differentiation than control moDCs (122). When examining the frequency of CD14^+^/CD16^+^ monocytes, conflicting results exist in severe asthmatics (123, 124). Monocytes of allergic patients showed increased IL-10 and decreased IL-12 production upon HDM and Der p1 stimulation, which enhanced Th2 differentiation (125). CD14^+^/CD16^+^ monocytes of severe asthmatics display higher expression of protease activation receptor 2 (PAR-2) as compared to mild/moderate asthmatics (124) (Figure 2B). PAR-2-mediated activation of monocytes induces secretion of IL-1β, IL-6, and IL-8 (126), indicating that activation *via* PAR-2 facilitates secretion of cytokines important for Th17-cell differentiation and neutrophil activation and attraction, which does not occur in mild/moderate asthmatics.

## Clinical Implications

In conclusion, whereas in mice the function of different DC subsets in asthma pathogenesis is becoming more and more clear, there are no studies at present that compared the Th2- or Th17-priming capacity of different human DC subsets in response to allergens. The limited number of DCs in peripheral blood and the difficulty to obtain lung or lung-draining lymph nodes hamper these studies. Current advances in single-cell analysis enable analysis of DC subsets and have already proven that more DC subsets and DC precursors can be found in peripheral blood (119, 120). Further research should provide insights into DC subset characteristics and function in asthmatics that display either a Th2, Th2/Th17, or Th17-mediated inflammation.

## Author Contributions

All authors listed have made a substantial, direct, and intellectual contribution to the work and approved it for publication.

## Conflict of Interest Statement

The authors declare that the research was conducted in the absence of any commercial or financial relationships that could be construed as a potential conflict of interest.

## Abbreviations

AHR, airway hyperresponsiveness; BAL, bronchoalveolar lavage; BATF3, basic leucine zipper ATF-like transcription factor 3; BM, bone marrow; C, complement; CCL, chemokine ligand; CCR, chemokine receptor; cDC, conventional dendritic cell; CDP, common DC progenitor; cMoP, common monocyte progenitors; CSF-1, colony-stimulating factor 1; DC, dendritic cell; ID-2, DNA-binding protein inhibitor 2; Der p1, Dermatophagoides pteronyssinus antigen P1; DTR, diphtheria toxin; Flt3L, FMS-like tyrosine kinase 3 ligand; GM-CSF, granulocyte macrophage colony-stimulating factor; HDM, house dust mite; HLA-DR, human leukocyte antigen-D; HSC, hematopoietic stem cell; ICOSL, inducible T-cell costimulator ligand; ID2, inhibitor of DNA binding 2; IFN-α, interferon alpha; IL, interleukin; IRF, interferon regulatory factor; MDP, macrophage DC progenitor; MLN, mediastinal lymph node; mDCs, myeloid DCs; moDC, monocyte-derived DC; OVA, ovalbumin; OX-40L, OX-40 ligand; PAR-2, protease-activated receptor 2; pDC, plasmacytoid dendritic cell; PD-L1, programmed death-ligand 1; PPARγ, peroxisome proliferator-activated receptor gamma; PU.1, hematopoietic transcription factor PU.1; RA, retinoic acid; RALDH, retinal dehydrogenase; RBPJ, recombination signal-binding protein 1 for J-Kappa; RELB, v-rel avian reticuloendotheliosis viral oncogene homolog B; STAT3, signal transducer and activator of transcription 3; Th, t helper; TLR, toll like receptor; TNFAIP3, tumor necrosis factor alpha interacting protein 3; Treg, regulatory T cell; TSLP, thymic stromal lymphopoietin.
